# Uncemented and cemented primary total hip arthroplasty in the Swedish Hip Arthroplasty Register: Evaluation of 170,413 operations

**DOI:** 10.3109/17453671003685400

**Published:** 2010-03-31

**Authors:** Nils P Hailer, Göran Garellick, Johan Kärrholm

**Affiliations:** ^1^Department of Orthopaedics, Institute of Surgical Sciences, Uppsala University HospitalUppsala; ^2^Department of Orthopaedics, Institute of Surgical Science, Sahlgrenska University Hospital, Göteborg University, MölndalSweden

## Abstract

**Background and purpose:**

Since the introduction of total hip arthroplasty (THA) in Sweden, both components have most commonly been cemented. A decade ago the frequency of uncemented fixation started to increase, and this change in practice has continued. We therefore analyzed implant survival of cemented and uncemented THA, and whether the modes of failure differ between the two methods of fixation.

**Patients and methods:**

All patients registered in the Swedish Hip Arthroplasty Register between 1992 and 2007 who received either totally cemented or totally uncemented THA were identified (n = 170,413). Kaplan-Meier survival analysis with revision of any component, and for any reason, as the endpoints was performed. Cox regression models were used to calculate risk ratios (RRs) for revision for various reasons, adjusted for sex, age, and primary diagnosis.

**Results:**

Revision-free 10-year survival of uncemented THA was lower than that of cemented THA (85% vs. 94%, p < 0.001). No age or diagnosis groups benefited from the use of uncemented fixation. Cox regression analysis confirmed that uncemented THA had a higher risk of revision for any reason (RR = 1.5, 95% CI: 1.4–1.6) and for aseptic loosening (RR = 1.5, CI: 1.3–1.6). Uncemented cup components had a higher risk of cup revision due to aseptic loosening (RR = 1.8, CI: 1.6–2.0), whereas uncemented stem components had a lower risk of stem revision due to aseptic loosening (RR = 0.4, CI: 0.3–0.5) when compared to cemented components. Uncemented stems were more frequently revised due to periprosthetic fracture during the first 2 postoperative years than cemented stems (RR = 8, CI: 5–14). The 5 most common uncemented cups had no increased risk of revision for any reason when compared with the 5 most commonly used cemented cups (RR = 0.9, CI: 0.6–1.1). There was no significant difference in the risk of revision due to infection between cemented and uncemented THA.

**Interpretation:**

Survival of uncemented THA is inferior to that of cemented THA, and this appears to be mainly related to poorer performance of uncemented cups. Uncemented stems perform better than cemented stems; however, unrecognized intraoperative femoral fractures may be an important reason for early failure of uncemented stems. The risk of revision of the most common uncemented cup designs is similar to that of cemented cups, indicating that some of the problems with uncemented cup fixation may have been solved.

## Introduction

During the 1970s, after about one decade of successfully performed cemented total hip arthroplasties (THAs), uncemented implants were introduced in increasing numbers. Different principles of fixation such as screw rings, press-fit with or without additional screws, and various new materials were used. With time, it became evident that these materials should have good biocompatibility and a specific surface structure to achieve secondary fixation to the bone, in order to avoid later loosening. Many implant designs not fulfilling these criteria turned out to be failures, unfortunately often in a large number of patients. When the problem of fixation seemed to be solved, wear and osteolysis reappeared also with uncemented cups and turned out to be even worse than observed for the cemented designs. Even today, the focus is on the articulating surfaces and materials with the hope that recent technology will solve these problems. Numerous uncemented implants with different appearances have indeed shown excellent results in small or medium-sized cohorts (Badhe et al. 2002, Gabbar et al. 2008, Reigstad et al. 2008, Aldinger et al. 2009, Gwynne-Jones et al. 2009), in contrast to older designs with unacceptable failure rates (Puolakka et al. 1999, Thanner et al. 1999, Lai et al. 2002).

In Sweden, the Swedish Hip Arthroplasty Register (SHAR) has well-documented low revision rates after cemented THA (87% survival after 17 years), and this prevented Sweden from swiftly introducing uncemented THA—which became frequently used in many other European countries and in North America. Although the use of uncemented THA has increased steadily and slowly for many years in Sweden, the proportion of uncemented THA relative to cemented THA is still low by international standards (Swedish Hip Arthroplasty Register, Annual Report 2007). Cemented THA remains the gold standard for older patients (> 65 yrs) and for almost all patients with cervical neck fractures, whereas uncemented THA is more commonly used in younger patients.

Our objective was to compare the outcome of uncemented and cemented THA in the SHAR. Specifically, we intended to investigate whether certain age groups or disease groups might benefit more than others from the use of uncemented THA, and whether modes of failure differ between the two fixation principles.

## Patients and methods

### Sources of data

Data were extracted from the Swedish Hip Arthroplasty Register (SHAR). Each patient receiving a primary or secondary THA is registered and reported by the operating unit using a personal identification number. This number is linked to relevant information such as change of address, date of emigration, or the date of death. The Swedish Hip Arthroplasty Register has been validated repeatedly (Söderman 2000, Swedish Hip Arthroplasty Register, Annual Report 2007). Since 1992, detailed information on demographics and implant specifications have been linked to the personal identification number, which enables more comprehensive and reliable studies of individual implant designs, as in this study. All units performing THA in Sweden report to the register and completeness on an individual basis is 98% for primary THA and 94% for revision THA.

### Study population

We identified all primary THAs registered in the SHAR between 1992 and 2007 that were operated with either totally cemented or totally uncemented fixation. Hybrid, inverse hybrid, and resurfacing arthroplasties were excluded. This left a study population of 145,339 patients with 170,413 THAs.

### Statistics

Follow-up started on the day of primary THA and ended on the day of revision, death, emigration, or December 31, 2007, whichever came first. Revision was defined as exchange or removal of any part of the cup or stem, or the entire implant.

Kaplan-Meier survival analyses were performed with the type of fixation as the independent factor, and revision of any component and for any reason as primary endpoints. Further analyses were performed after stratification according to age group (< 50, 50–59, 60–75, > 75 years), or after stratification according to diagnosis group (primary osteoarthritis (OA), inflammatory hip disease (e.g. rheumatoid arthritis, M. Bechterew), femoral neck fracture, childhood hip disease, idiopathic femoral head necrosis, secondary posttraumatic OA, tumor). The log-rank test (Mantel-Cox) was used to investigate whether there was a significant difference between groups, and p < 0.05 was chosen as the predetermined level of significance.

In order to adjust for possible confounding factors, a Cox proportional hazards model was used to analyze the relative risk (RR) of revision with 95% confidence intervals (CIs). Endpoints were revision of any component (1) for any reason, (2) due to infection, (3) due to aseptic loosening, or (4) due to periprosthetic fracture. We hypothesized that the reasons for revision might differ between uncemented and cemented THA during the early phase after the index procedure; thus, we performed a separate analysis with revision for any reason within 2 years of the index procedure, or with revision due to periprosthetic fracture within 2 years of the index procedure as the endpoint. In further analyses, the endpoint was revision of either cup or stem for the reasons described above. In a separate set of analyses, the 5 most commonly used implants in each group were compared with each other: the 5 most common cemented cups (Lubinus, Charnley, Exeter Duration, Charnley Elite, and Reflection) with the 5 most common uncemented cups (Trilogy HA, CLS Spotorno, Trilogy, Trident HA, and Allofit), and the 5 most common cemented stems (Lubinus SP2, Exeter polished, Charnley, Spectron EF Primary, and Scan Hip Collar) with the 5 most common uncemented stems (CLS, Bi-Metric HA, ABG, Omnifit, and Wagner Cone). The covariates type of fixation (cemented or uncemented), sex, age (stratified into the 4 age groups described above), and primary diagnosis before THA surgery (see above) were entered into the model. An initial analysis was performed where all covariates mentioned above were entered as singular variables and a crude risk ratio was calculated for each variable. Thereafter, all covariates mentioned above were entered in the regression model and risk ratios were mutually adjusted for all covariates.

The assumption of proportional hazards was investigated and verified by hazard-function plots that never crossed or approximated each other in any analysis, and by log-minus-log plots that ran strictly parallel in all analyses. It has been pointed out that the inclusion of both THAs in bilaterally operated patients can create dependency problems (Ranstam 2002), and we therefore investigated whether our Cox regression model was robust against this potential violation. Separate analyses were done either after including all THAs (170,413 THAs in 145,339 individuals) or after excluding the second side in bilaterally operated patients (leaving 145,339 THAs in 145,339 individuals). The calculated adjusted risk ratios were not statistically significantly affected by the inclusion of both THAs in bilaterally operated patients (data not shown), which is in accordance with previously published findings (Lie et al. 2004, Thillemann et al. 2008). Statistical analyses were performed using SPSS (version 16.0), except for the calculation of life tables, which was carried out with SAS (version 9.2).

## Results

### Characteristics of the study population

There were more females than males in the study population, and primary osteoarthritis was the most common preoperative diagnosis. Most patients were in the 60–75-year age group (Table 1). Cemented components were chosen in 161,460 procedures; 8,953 were totally uncemented. The 10 most commonly used cup and stem implants in the two groups are described in Table 2. By 2007, 6,636 (3.9%) of all 170,413 arthroplasties had been revised, mostly due to aseptic loosening (2.3%), dislocation (0.7%), or deep infection (0.5%). The mean observation time was 5.9 years (SD 4) for the group of cemented THAs and 4.9 years (SD 4.5) for the group of uncemented THAs.

**Table 1.** Patient demographics

	n	%
Sex		
Male	56,532	39
Female	88,805	61
Age (years)		
0–49	5,182	4
50–59	16,182	11
60–75	74,950	52
> 75	49,025	34
Primary diagnosis		
Primary OA	110,438	76
Inflammatory disease	5,397	4
Fracture	18,832	13
Pediatric hip disease	2,073	1
Idiopathic femoral head necrosis	4,430	3
Secondary posttraumatic OA	371	0.3
Others		1

**Table 2.** The 10 most common implants

	n	%
A) The 10 most common cemented cups		
Lubinus	62,044	38
Charnley	28,386	18
Exeter Duration	11,938	7
Charnley Elite	11,351	7
Reflection	8,351	5
Exeter Plast	6,668	4
FAL	4,513	3
Biomet Müller	4,099	3
Scan Hip Cup	3,937	2
OPTICUP	3,779	2
Other		2
B) The 10 most common cemented stems		
Lubinus SP II	69,991	43
Exeter Polished	33,347	21
Charnley	23,649	15
Spectron EF Primary	8,093	5
Scan Hip Collar	2,989	2
Charnley Elite Plus	2,716	2
Scan Hip II Collar	2,229	1
Müller Straight Stem	2,165	1
Bi-Metric cemented	1,529	1
MS30 Polished	1,509	1
Other		8
C) The 10 most common uncemented cups		
Trilogy HA	1,856	21
CLS Spotorno	1,026	12
Trilogy	868	10
Trident HA	770	9
Allofit	725	8
ABG II HA	433	5
Romanus	405	5
Romanus HA	351	4
Omnifit	325	4
ABG HA	287	3
Other		21
D) The 10 most common uncemented stems		
CLS Spotorno	3,572	40
Bi-Metric HA uncemented	729	8
ABG uncemented	492	6
Omnifit	482	5
Wagner Cone Prosthesis	370	4
Accolade	369	4
Bi-Metric uncemented	334	4
ABG II HA	313	4
Bi-Metric lateral	287	3
Versys	263	3
Other		20

### Revision risk—all cemented vs. all uncemented

Kaplan-Meier survival analysis showed that revision-free component survival at 10 years was lower for uncemented THA than for cemented THA when revision of any component and for any reason was the endpoint (85% vs. 94%, p < 0.001 (Figure). At 15 years, survival dropped to 70% (CI: 67–73) in the group of uncemented THAs and to 88% (CI: 88–89) in the group of cemented THAs. After 15 years, there were 245 THAs at risk in the group of uncemented THAs whereas 3,147 THAs were at risk among the cemented THAs.

**Figure:**
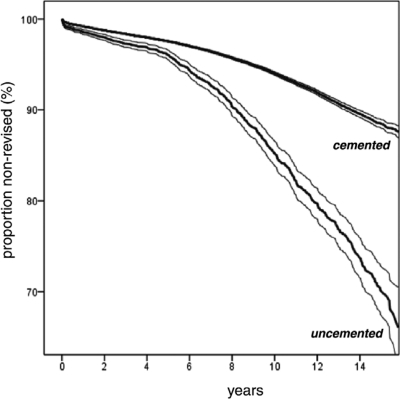
Kaplan-Meier analysis of survival of cemented and uncemented THA. Revision-free survival was significantly lower for uncemented THA than for cemented THA with revision of any component for any reason as endpoint. Bold lines: survival: thin lines: upper and lower limits of 95% confidence intervals. 10-year-survival: 85% (CI: 84–87) for uncemented THA vs. 94% (CI: 93.8–94.2) for cemented THA (p < 0.05, log-rank test). 15-year-survival: 70% (CI: 67–73; 245 THAs at risk) for uncemented THA vs. 88% (CI: 88–89; 3,147 THAs at risk) for cemented THA (p < 0.05, log-rank test).

After stratification of the study population into age groups, we found that component survival with revision for any reason as the endpoint was lower after uncemented THA in all but the oldest age group (p < 0.05 for the strata < 50, 50–59, and 60–75 years). Stratification of the study population into groups of diagnoses showed that uncemented THA had lower survival in nearly all groups, i.e. primary OA, rheumatoid arthritis, femoral neck fracture, childhood hip disease, idiopathic femoral head necrosis, and secondary posttraumatic OA (p < 0.05 in these diagnosis groups).

The Cox regression model showed that uncemented THA had a higher risk of revision for any reason than cemented THA (RR = 1.5, CI: 1.4–1.6) after adjustment for the covariates sex, age, and underlying diagnosis (Table 3 and Table 6). Uncemented THA also had a significantly higher risk of revision due to aseptic loosening (RR = 1.5, CI: 1.3–1.6) (Table 4 and Table 6). In contrast, uncemented THA did not have a higher risk of revision due to infection than cemented THA (RR = 0.9, CI: 0.6–1.3) (Table 5 and Table 6).

**Table 3.** Cox regression model with relative risk (RR) of revision for any reason.The covariates type of fixation, sex, age, and underlying diagnosis were mutually adjusted for each other. Revision means revision of either cup or stem, or both

	Adjusted RR	CI	p-value
Type of fixation			
Cemented **^a^**	1		
Uncemented	1.5	(1.4 –1.6)	< 0.001
Sex			
Male **^a^**	1		
Female	0.7	(0.6 –0.7)	< 0.001
Age			
< 50 **^a^**	1		
50–59	0.8	(0.7 –0.9)	< 0.001
60–75	0.5	(0.5 –0.6)	< 0.001
> 75	0.4	(0.4 –0.4)	< 0.001
Diagnosis			
Primary OA **^a^**	1		
RA	1.2	(1.1 –1.4)	< 0.001
Fracture	1.9	(1.7 –2.0)	< 0.001
Pediatric	1.6	(1.4 –1.8)	< 0.001
Osteonecrosis	1.5	(1.3 –1.7)	< 0.001
Posttraumatic OA	2.1	(1.6 –2.8)	< 0.001
Tumor	2.3	(1.5 –3.5)	< 0.001
Other secondary arthrosis	1.0	(0.7 –1.3)	0.7
**^a^** Reference group that other categories are compared to			

**Table 4.** Cox regression model with relative risk (RR) of revision due to aseptic loosening. The covariates type of fixation, sex, age, and underlying diagnosis were mutually adjusted for each other. Revision means revision of either cup or stem, or both

	Adjusted RR	CI	p-value
Type of fixation			
Cemented **^a^**	1		
Uncemented	1.5	(1.3 –1.6)	< 0.001
Sex			
Male **^a^**	1		
Female	0.7	0.6 –0.7)	< 0.001
Age			
< 50 **^a^**	1		
50–59	0.7	(0.6 –0.8)	< 0.001
60–75	0.4	(0.4 –0.5)	< 0.001
> 75	0.2	(0.2 –0.2)	< 0.001
Diagnosis			
Primary OA **^a^**	1		
RA	1.1	(1.0 –1.2)	0.2
Fracture	1.2	(1.1 –1.4)	0.02
Pediatric	1.5	(1.3 –1.8)	< 0.001
Osteonecrosis	1.3	(1.1 –1.5)	0.01
Posttraumatic OA	1.5	(1.0 –2.2)	0.05
Tumor	1.4	(0.5 –3.7)	0.5
Other secondary arthrosis	0.7	(0.4 –1.0)	0.03
**^a^** Reference group that other categories are compared to			

**Table 5.** Cox regression model with relative risk (RR) of revision due to infection. The covariates type of fixation, sex, age, and underlying diagnosis were mutually adjusted for each other. Revision means revision of either cup or stem, or both

	Adjusted RR	CI	p-value
Type of fixation			
Cemented **^a^**	1		
Uncemented	0.9	(0.6 –1.3)	0.5
Sex			
Male **^a^**	1		
Female	0.5	(0.4 –0.5)	< 0.001
Age			
< 50 **^a^**	1		
50–59	0.8	(0.6 –1.2)	0.4
60–75	0.7	(0.5 –1.1)	0.1
> 75	0.7	(0.5 –1.0)	0.07
Diagnosis			
Primary OA **^a^**	1		
RA	1.4	(1.0 –1.9)	0.04
Fracture	1.8	(1.4 –2.2)	< 0.001
Pediatric	1.2	(0.7 –2.0)	0.6
Osteonecrosis	1.4	(0.9 –2.0)	0.1
Posttraumatic OA	0.5	(0.1 –3.6)	0.5
Tumor	1.7	(0.5 –5.3)	0.4
Other secondary arthrosis	1.1	(0.5 –2.5)	0.8
**^a^**Reference group that other categories are compared to.			

**Table 6.** Summary of adjusted risk ratios. Revision during the entire study period

Endpoint	Population investigated	Reference population	Adjusted RR (CI)	p-value
Revision of any component				
for any reason	All uncemented implants	All cemented implants	1.5 (1.4–1.6)	< 0.001
due to aseptic loosening	All uncemented implants	All cemented implants	1.5 (1.3–1.6)	< 0.001
due to deep infection	All uncemented implants	All cemented implants	0.9 (0.6–1.3)	0.5
for any reason	All uncemented implants from 2000	All cemented implants from 2000	1.5 (1.2–1.8)	< 0.001
Cup revision due to aseptic loosening	All uncemented cups	All cemented cups	1.8 (1.6–2.0)	< 0.001
Stem revision due to aseptic loosening	All uncemented stems	All cemented stems	0.4 (0.3–0.5)	< 0.001
Revision for any reason within 2 years of the index procedure	All uncemented implants	All cemented implants	1.8 (1.5–2.2)	< 0.001
Stem revision due to fracture within 2 years of the index procedure	All uncemented stems	All cemented stems	8.0 (4.5–14)	< 0.001
Cup revision for any reason	5 most common uncemented cups	5 most common cemented cups	0.9 (0.6–1.1)	0.2
Cup revision due to aseptic loosening	5 most common uncemented cups	5 most common cemented cups	0.5 (0.3–0.8)	0.001
Stem revision for any reason	5 most common uncemented stems	5 most common cemented stems	0.5 (0.4–0.6)	< 0.001
Stem revision due to aseptic loosening	5 most common uncemented stems	5 most common cemented stems	0.3 (0.2–0.4)	< 0.001

### Revision risk of cup and stem analyzed separately

Uncemented cups had a significantly higher risk of cup revision due to aseptic loosening than cemented cups (RR = 1.8, CI: 1.6–2.0) after adjustment for sex, age, and diagnosis (Table 6). This difference also persisted after exclusion of revisions where only liner exchanges had been performed (RR = 1.5, CI: 1.4–1.7).

In contrast, after adjustment for sex, age, and underlying diagnosis, uncemented stem components had a lower risk of stem revision due to aseptic loosening than cemented stems (RR = 0.4, CI: 0.3–0.5) (Table 6).

### Revision risk within 2 years of the index procedure

A Cox regression model with revision within 2 years of the index procedure (of any component and for any reason) as the endpoint showed that uncemented arthroplasties had a higher risk of revision than cemented arthroplasties during this early time period (RR = 1.8, CI: 1.5–2.2). The reasons for revision within 2 years differed markedly between the two groups. The main finding was that a much higher proportion of uncemented THAs were revised due to periprosthetic fracture during the first 2 years, 17% in the group of uncemented THAs versus 6% in the group of cemented THAs. Cox regression analysis adjusting for sex, age, and underlying diagnosis showed that the risk of stem revision due to periprosthetic fracture within 2 years of the index procedure was remarkably high when comparing uncemented and cemented THAs (RR = 8, CI: 5–14) (Table 6).

### Revision risk of commonly used cups and stems

The risk of revision for various reasons was also calculated after selection of THAs where the 5 most commonly used implants in each group had been used. It became apparent that the risk of cup revision for any reason was similar between the 5 most commonly used cemented cups and the 5 most commonly used uncemented cups (RR for uncemented vs. cemented cups = 0.9, CI: 0.6–1.1). With cup revision due to aseptic loosening as the endpoint, the risk of revision was lower in the group of 5 most commonly used uncemented cups compared with the group of 5 most commonly used cemented cups (RR = 0.5, CI: 0.3–0.8) (Table 6). The mean observation time was 3.2 years (SD 3) for the group of 5 most common uncemented cups and 5.8 years (SD 4) for the group of 5 most common cemented cups.

The risk of stem revision for any reason was lower for the 5 most commonly used uncemented stems than for the 5 most commonly used cemented stems (RR = 0.5, CI: 0.4–0.6) (Table 6). With the risk of stem revision due to aseptic loosening as the endpoint of the analysis, it was found that the 5 most commonly used uncemented stems again had a lower risk of revision than the 5 most commonly used cemented stems (RR = 0.3, CI: 0.2–0.4; Table 6). The mean observation time was 5.1 years (SD 4.3) for the group of 5 most common uncemented stems and 5.8 years (SD 4) for the group of 5 most common cemented stems.

Because the design of non-cemented hip arthroplasty components has been altered since the introduction of uncemented fixation, and because surgical experience with uncemented implants has increased over time, we thought it possible that contemporary uncemented THA would show improved component survival compared to older devices and compared to devices implanted early after the introduction of uncemented THA in Sweden. We therefore performed the same analyses as described above, but restricted data selection to all hips that were operated from the year 2000 onward. The risk of revision of any component for any reason was still higher for uncemented THA than for cemented THA (RR = 1.5, CI: 1.2–1.8) (Table 6).

## Discussion

The Swedish experience of inferior results with uncemented THA contrasts with the general conception of this type of fixation (Morshed et al. 2007). Nonetheless, the use of uncemented fixation is increasing even in Sweden, for unknown reasons. Factors such as shorter operation time, good documentation of some uncemented designs in the SHAR and the Norwegian arthroplasty register, and large cohort studies may have stimulated this development.

We found that no age group or diagnosis group benefited from the choice of uncemented THA, which conflicts with findings on the superior outcome of uncemented arthroplasty in younger groups of patients (Eskelinen et al. 2006, Hooper et al. 2009). This is also contrasted by research on the biology of osseointegration of uncemented implants, where the potential of implant integration into host bone has been used as an argument for uncemented fixation (Willert and Buchhorn 1999). On the basis of our findings, there is nothing to support the notion that younger patients with a diagnosis of primary osteoarthritis can expect better revision-free survival after uncemented fixation than after cemented fixation.

Separate analyses of the cup and stem components revealed that they contributed to the risk of revision in different ways. In uncemented THA, the acetabular cup appeared to be the component that was associated with increased risk of revision, irrespective of whether revision for any reason or due to aseptic loosening was considered. In contrast, uncemented stems performed better than cemented stems, again irrespective of the chosen endpoint of analysis.

The observation that uncemented fixation of metal cups provides inferior long-term results could be attributed to an increased risk of wear-related problems. Because the risk of revision persisted after exclusion of isolated liner exchanges in our analyses, it seems reasonable to believe that osteolysis and wearing-through of the liner, with secondary damage to the shell, were important reasons for revision. Several studies have indicated that an isolated liner exchange increases the risk of further revisions, and not least increases the risk of dislocation (Lie et al. 2007). This may have stimulated a more active attitude among Swedish surgeons to also extract the shell in situations of revision due to liner wear. However, according to numerous studies, migration and loosening of modern press-fit cup designs is rare (Thanner et al. 2000, Rohrl et al. 2006). In the SHAR, loosening, wear, and osteolysis are not primarily separated as causes of revision.

The question of whether the superior long-term results of some monobloc cup designs, where the polyethylene has been molded into various metal cases are superior, cannot be answered in this study, because none of these cups has been in frequent use in Sweden. As for many other contemporary designs and articulations, mostly medium-term results are available for monobloc cups (Hinrichs et al. 2001, Unger et al. 2005, Malizos et al. 2008, Gwynne-Jones et al. 2009). However, problems due to osteolysis and wear rarely result in revision surgery until 6–8 years have elapsed; thus, optimistic reports on the survival of monobloc cups must be regarded with some caution.

Detailed information on the type of polyethylene was not available for our analysis; therefore, inferior performance of certain types of polyethylene that were predominantly used in uncemented cups may have influenced our findings, but we do not believe that this accounts for the overall inferior performance of uncemented cups. Alternative bearing types such as metal-on-metal, ceramic-on-polyethylene, and ceramic-on-ceramic have not been in widespread use in Sweden; thus, various problems associated with these bearing types should not have distorted our findings.

In contrast to the inferior performance of uncemented cups, we found superior survival of uncemented stems compared to cemented stems. This finding could at least in part be explained by the fact that cemented stems of smaller sizes, i.e. mostly stems inserted into narrow femora, have an increased risk of revision, perhaps due to thin or absent cement mantles (Swedish National Hip Arthroplasty Register, Annual Report 2005). We also found that the risk of early revision due to fracture was much higher for uncemented stems than for cemented stems. It could be proposed that this finding indicates that patients who receive uncemented arthroplasty are more vulnerable to trauma during the first postoperative year, although the fact that the risk ratio has been adjusted for age, sex, and underlying diagnosis in the Cox regression model contradicts this proposal. It seems more probable that some of these fractures occur during the index operation but remain undetected at the time of surgery. In some cases, minor fissures—invisible on ordinary radiographs—may have been produced during stem insertion, which may explain why these patients suffer a manifest periprosthetic fracture even after minor trauma. This hypothesis is supported by reports of a relatively high incidence of intraoperative fractures associated with stem insertion during uncemented THA (Fernandez-Fernandez et al. 2008).

The issue of periprosthetic fractures is probably related to general surgical experience, and to implant-specific learning curves. It could be argued that cemented arthroplasty is a more forgiving technique than uncemented arthroplasty, although the finding of high revision rates after improperly performed cementation indicates that cemented THA has its own pitfalls. The SHAR has not been designed to investigate single-surgeon outcomes; the aspect of learning curves cannot therefore be analyzed in detail. It does, however, seem reasonable to assume that theoretical and practical education of surgeons who are introduced to uncemented THA should be able to reduce the risk of complications mentioned above.

It has been proposed that hip arthroplasties fixed with antibiotic-laden cement should be less prone to deep infection than cemented implants fixed with conventional cement that contains no additional antibiotics (Engesaeter et al. 2003, Parvizi et al. 2008). In the SHAR, more than 90% of the cases were cemented with antibiotic-laden cement, but further analysis of this factor is uncertain because the use of cement has often been reported with data aggregated for each participating unit. Regarding the risk of deep infection, to our knowledge no other direct comparison of large groups of patients receiving either cemented or uncemented arthroplasty has been published. Thus, it appears that the risk of revision due to infection should be about equal if uncemented fixation is compared with cemented fixation, provided that the cement is antibiotic-laden.

In a separate analysis, we investigated the risk of revision of commonly used uncemented or cemented cup and stem designs. We found that the most commonly used uncemented cups did not show any increased risk of revision for any reason when compared with the most commonly used cemented cups. When revision due to aseptic loosening was considered, common uncemented cups even seemed to perform better than common cemented cups. On the other hand, the mean time of observation of uncemented cups was shorter than that of cemented cups; thus, future problems of wear and osteolysis could distort this finding. When commonly used uncemented stems were compared with commonly used cemented stems, the previously described advantage of uncemented stems observed in the entire study cohort was confirmed: the group of commonly used uncemented stems had a lower risk of revision for any reason and a lower risk of revision due to aseptic loosening than commonly used cemented stems. We therefore believe that our analysis is not unfavorably biased against uncemented THA itself, but that our findings highlight that at least some of the difficulties associated with early uncemented implant fixation have been recognized and reduced in the uncemented designs that now prevail on the Swedish market.

Several weaknesses must be kept in mind during the interpretation of our data: (1) all registry studies suffer from specific uncertainties concerning data collection, reliability, and validity, and this also applies to the present material. However, in the light of previously performed validations of the SHAR, we believe that the error margins are small and do not substantially distort the basic findings of our study (Söderman 2000); (2) a further potential weakness is the large variation in the choice of implants. Variations in the type of implant were larger among uncemented components than among cemented components: the 10 most common cemented cups and stems made up more than 90% of all implants, whereas the 10 most common uncemented implants only covered 80% of all components. Thus, the group of uncemented THAs was more heterogeneous, and a number of uncemented designs with inferior performance—although not very widely used or even out of current use—could distort the performance of the group of uncemented THAs as a whole. However, some implants with known catastrophic results, such as the ABG I cup, were not in widespread use in Sweden during the study period and should therefore not have negatively biased the group of uncemented cups as a whole (Blacha 2004). It remains to be seen whether future use of designs with superior survival will improve the general performance of uncemented THAs.

A registry study such as ours cannot be compared directly with the results of a defined implant in the hands of a dedicated, often small group of surgeons—such as in most small or medium-sized cohort studies. Our study rather reflects the nationwide outcome of many different implants in many different hands, with surgeons who are more or less experienced in the technique of uncemented THA surgery. It would therefore be over-hasty to condemn uncemented THA as a whole, but a few words of caution should suffice: (1) uncemented cups are the Achilles’ tendon of uncemented THA. It remains to be seen whether other types of cup or liner fixation, cup coating, or more wear-resistant surface bearings can reduce the problem of cup loosening; (2) the risk of stem revision due to fracture during the first 2 years is approximately 8-fold higher for uncemented stems than for cemented stems. This indicates that a number of perioperatively induced femoral fissures remain unrecognized, which is probably a question of surgical technique. The need for proper training of surgeons who start using uncemented implants is highlighted by these findings. Our study does not support the notion that uncemented THA is superior to cemented THA when looking at long-term implant survival. At best, some of the common contemporary designs can reach an outcome that equals that of cemented THA.
